# Predicting the outcome of a cognitive-behavioral group training for patients with unexplained physical symptoms: a one-year follow-up study

**DOI:** 10.1186/1471-2458-12-848

**Published:** 2012-10-08

**Authors:** Lyonne NL Zonneveld, Yanda R van Rood, Cornelis G Kooiman, Reinier Timman, Adriaan van ’t Spijker, Jan JV Busschbach

**Affiliations:** 1 Department of Medical Psychology and Psychotherapy, Erasmus Medical Center, PO Box 2040, Rotterdam, 3000 CA, the Netherlands; 2 Department of Medical Psychology, Academic Medical Center, PO Box 22660, Amsterdam, 1100 DD, the Netherlands; 3 Department of Anesthesiology, Academic Medical Center, PO Box 22660, Amsterdam, 1100 DD, the Netherlands; 4 Department of Psychiatry, Leiden University Medical Centre, PO Box 9600, Leiden, 2300 RC, the Netherlands; 5 Riagg Rijnmond, Stationsplein 2, Schiedam, 3112 HJ, the Netherlands

**Keywords:** Predictors, Treatment outcome, Somatoform disorders, Unexplained physical symptoms, Cognitive behavioral therapy, Assessment

## Abstract

**Background:**

Although Cognitive-Behavioral Therapy (CBT) is effective for Unexplained Physical Symptoms (UPS), some therapists in clinical practice seem to believe that CBT outcome will diminish if psychiatric comorbidity is present. The result is that patients with a psychiatric comorbidity are redirected from treatment for UPS into treatment for mental health problems. To explore whether this selection and allocation are appropriate, we explored whether CBT outcomes in UPS could be predicted by variables assessed at baseline and used in routine-practice assessments.

**Methods:**

Patients (n=162) with UPS classified as undifferentiated somatoform disorder or chronic pain disorder were followed up until one year after they had attended a CBT group training. The time-points of the follow-up were at the end of CBT (immediate outcome), three months after CBT (short-term outcome), and one year after CBT (long-term outcome).

CBT outcome was measured using the Physical Component Summary of the SF-36, which was the primary outcome measure in the randomized controlled trial that studied effectiveness of the CBT group training. Predictors were: 1.) psychological symptoms (global severity score of SCL-90), 2.) personality-disorder characteristics (sum of DSM-IV axis II criteria confirmed), 3.) psychiatric history (past presence of DSM-IV axis I disorders), and 4.) health-related quality of life in the mental domain (mental component summary of SF-36). The effect of this predictor set was explored using hierarchical multiple regression analyses into which these predictors had been entered simultaneously, after control for: a.) pretreatment primary outcome scores, b.) age, c.) gender, d.) marital status, and e.) employment.

**Results:**

The predictor set was significant only for short-term CBT outcome, where it explained 15% of the variance. A better outcome was predicted by more psychological symptoms, fewer personality-disorder characteristics, the presence of a psychiatric history, and a better quality of life in the mental domain.

**Conclusions:**

As the predictors do not seem to predict CBT outcome consistently over time, the need for selection and allocation of patients for CBT is doubtful. It seems that this would unnecessarily deprive patients of effective treatment.

**Trial registration:**

Nederlands Trial Register, NTR1609

## Background

Although Cognitive-Behavioral Therapy (CBT) is effective for Unexplained Physical Symptoms (UPS) [1-6], some therapists in clinical practice seem to believe that it is not equally effective for all patients with UPS. Instead, they assume that outcome will be poorer in patients whose quality of life may have been affected by a psychiatric comorbidity such as depression, anxiety disorder, personality-disorder, or their psychiatric history.

Inconsistent findings have been produced by studies that investigated whether such comorbidity did indeed predict poor outcome [7-21]. Some studies showed that poor treatment outcome for UPS was predicted by concurrent depressive symptoms [9], anxiety symptoms [10], personality-disorder characteristics [11], a psychiatric history [12,21] or poor health-related quality of life [12]. Other studies used the same predictors to conclude differently. Thus, for concurrent depressive symptoms, one study found that depressive symptoms predicted a better outcome [13], while others showed no influence [7,8,10,11,14-20]. For concurrent anxiety symptoms, another study found that anxiety, too, predicted a better outcome [14], while others showed no influence [7,8,11,15-19]. For concurrent personality disorders, two further studies found that a personality disorder did not predict outcome – though they also implied that a personality disorder might increase the drop-out rate [8,16]. For psychiatric history, a further study showed no influence on outcome [9]. For health-related quality of life, another study reported that poorer functioning valued by assessors and a poorer quality of life reported by patients were associated with better outcome [15]. Conclusions on whether psychiatric comorbidity predicted outcome differed not only between studies, but also within them [12,15]. For example, one study [12] found that depressive symptoms did not predict post-treatment outcome, but did predict better three-month follow-up outcome.

One possible reason for these inconsistent findings on predicting CBT outcome in UPS is that the impact of psychiatric comorbidity was blurred by differences in the outcome scores at baseline or in socio-demographic variables at baseline. Various studies showed that the outcome was influenced by the pretreatment score on the outcome measure [10,12,15], and by socio-demographic variables such as age [10,12,14,20], gender [15], marital status [13], or having paid work [15,17].

To find predictors that consistently predict CBT outcome over time, we explored whether psychological symptoms, personality-disorder characteristics, psychiatric history, and health-related quality of life in the mental domain assessed at baseline predicted CBT outcome on the primary outcome measure at the end of CBT (immediate outcome), three months after CBT (short-term outcome), and one year after CBT (long-term outcome), all after control for pretreatment scores on the outcome measure and for socio-demographic variables. In line with clinical practice, that was supported but also contradicted by a number of studies, we hypothesized that better CBT outcome would be predicted by the following: fewer psychological symptoms and personality-disorder characteristics, the absence of a psychiatric history, and a better quality of life in the mental domain.

## Methods

### Design

The data for this study came from a randomized controlled trial on the effectiveness of cognitive-behavioral group training (CBT) for patients with UPS [22]. Patients with UPS were randomized either to CBT or to a waiting list after they had completed the baseline measurement (T0). The second measurement (T1) was made directly after the training (13 weeks), or, for those on the waiting list, after the same period. The maintenance of the effect of the group training was investigated in a non-randomized one-year follow-up. To this end, patients who had been randomized to the waiting list and had waited started the training after their second measurement (T1). Patients who had attended the training directly after randomization or after the waiting period were followed-up three months after the end of treatment (T2), and again one year later (T3).

The study was approved by the Erasmus Medical Research Ethics Committee, and registered in the Dutch Trial Register (NTR 1609) [23]. A detailed description of the protocol has been published earlier in this journal [24].

### Participants

Patients were recruited between February 2005 and September 2008 in general practices, in outpatient clinics at general hospitals, and by Riagg Rijnmond, a secondary community mental-health service for the greater Rotterdam area in the Netherlands. General practitioners and specialists were asked to refer patients aged between 18 and 65 years whose physical symptoms, according to clinical judgment, could not be fully explained on the basis of a known medical condition. Patients were included if they signed the informed consent and if their UPS fulfilled the DSM-IV criteria for an undifferentiated somatoform disorder or a chronic pain disorder.

We chose UPS classified with DSM-IV as ‘undifferentiated somatoform disorder’ or as ‘chronic pain disorder’, as these disorders were given clinical relevance by their high prevalence – in general practices, they are the most prevalent of all somatoform disorders [25] – and as they could be selected by valid and reliable instruments. ‘Undifferentiated somatoform disorder’ and ‘chronic pain disorder’ are non-overlapping disorders because of criterion E in the DSM-IV criteria for ‘undifferentiated somatoform disorder’, which states that the disorder can be assigned only if the symptoms are not better accounted for by another mental disorder such as another somatoform disorder.

To verify whether UPS fulfilled all DSM-IV criteria for either ‘undifferentiated somatoform disorder’ or ‘chronic pain disorder’, we used the Structured Clinical Interview for DSM-IV Axis I Disorders (SCID-I) [26], a semi-structured validated and reliable interview for making the major DSM-IV Axis I diagnoses.

Patients were excluded if they did not provide informed consent, or if poor language skills or handicaps such as cognitive impairment prevented them from understanding the CBT group training.

### CBT group training

The intervention, a CBT group training called ‘Coping with the consequences of unexplained physical symptoms’, is a weekly two-hour manual-based [27] training that is held over a 13-week period. It uses the following CBT techniques: psychoeducation, response prevention, pacing activity, graded activity, graded exercise, problem-solving, breathing and relaxation exercise, cognitive intervention using the Ellis’ ABC worksheet, and relapse prevention. Its aim is to improve health-related quality of life. A more detailed description of this CBT has been published elsewhere [24,28].

### CBT outcome measurement

In the randomized controlled trial, the primary outcome was the summary scales of the 36-item Medical Outcomes Study Short-Form General Health Survey (SF-36) [29]: Physical Component Summary’ (PCS) and ‘Mental Component Summary’ (MCS). In the present study, the PCS was chosen as outcome measurement, because patients reported the quality of life in the physical domain as most burdensome. The group training significantly improved quality of life in the physical domain, and this positive effect was maintained during the entire one-year follow-up period [30].

The PCS summarizes functional health and well-being in the physical domain over the past four weeks. This summary is transformed into T-scores with a mean of 50 and standard deviation of 10. A higher summary score indicates a better quality of life. The CBT outcome score was the difference between the baseline PCS score and the following: post-treatment PCS scores (immediate outcome); three-month follow-up PCS scores (short-term outcome); and one-year follow-up PCS scores (long-term outcome). A higher CBT outcome score indicates more improvement of quality of life in the physical domain.

### Predictors

#### Psychological symptoms

Psychological symptoms were measured using the *revised 90-item Symptom Checklist* (SCL-90-R). This is a validated and reliable self-report questionnaire with 90 questions and five fixed-response alternatives (Likert-type format: Not at all; Somewhat; Moderately; Very much; Absolutely) for evaluating a broad range of psychological symptoms, including anxiety and depression, over the past week [31]. The responses are summed up in the ‘Global severity index’. A higher score on this index indicates more severe psychological symptoms or a higher number of psychological symptoms.

#### Personality-disorder characteristics

Personality-disorder characteristics were measured using the *Vragenlijst Kenmerken van Persoonlijkheid* (VKP), a Dutch self-report questionnaire based on the International Personality Disorder Examination [32]. The VKP is a validated and reliable self-report questionnaire with 197 questions and three fixed-response alternatives (true; ?; false) for assessing the presence of DSM-IV axis II criteria of personality disorders over the past five years. ‘Personality-disorder characteristics’ were calculated by summing DSM-IV axis II criteria, to which was responded with “true”. A higher sum score indicates a higher number of DSM-IV axis II criteria confirmed.

#### Presence of DSM-IV axis I disorders in the past (‘psychiatric history’)

The presence per patient of DSM-IV axis I disorders, both currently and over their lifetime, was measured using the *Structured Clinical Interview for DSM-IV axis I disorders* (SCID-I) [26]. This is a semi-structured validated interview for classifying the major DSM-IV axis I disorders. The presence of these disorders in the past (‘psychiatric history’) was calculated by summing disorders in lifetime that were not currently present, and splitting the sum score into two categories (no DSM-IV axis I in the past, 0; or one or more DSM-IV axis I disorders in the past that were not currently present, 1).

#### Health-related quality of life in the mental domain (‘mental component summary’)

Health-related quality of life in the mental domain was measured using the ‘Mental Component Summary’ (MCS) of the SF-36 [29]. The MCS summarizes functional health and well-being in the mental domain over the past four weeks. This summary is transformed into T-scores with a mean of 50 and standard deviation of 10. A higher MCS-score indicates a better health-related quality of life in the mental domain.

#### Control variables

Pretreatment PCS scores, age, gender, marital status and employment status were used as control variables.

### Statistical analyses

#### Required sample size

The randomized controlled trial on the effectiveness of the CBT group training resulted in a group of 162 patients. To verify whether this fixed number of patients was also sufficient for the present study, we applied a power analysis to calculate the sample size required for the present study [33,34].

For this power analysis, the anticipated effect size of the set predictors was set at f^2^=0.15 [35]. We decided that the set should at least have this medium effect, because the predictors would exclude patients from treatment that had an exceptionally small risk of adverse events [36], and, also, because the selection and allocation assessment needed for this exclusion would raise costs. The desired statistical power level was set at 0.80 and the alpha at 0.05; both by convention [35]. The number of predictors was four, while the number of control variables was five. The predictors were selected on the basis of assumptions practiced in clinical practice. The control variables were chosen on the basis of findings of other studies that indicated the potential relevance of these variables for CBT outcome. By selecting predictors used in clinical practice and by choosing control variables indicated by studies as potentially relevant, we reduced the number of predictors and control variables, and prevented ‘fishing’.

A power analysis with these parameters led to a minimum required sample size of 113 patients [37,38]. Adjusted for a dropout of 30 percent, this resulted in a total sample size of 161. The total sample size of 162 in the randomized controlled trial was thus sufficient for the hierarchical multiple regression analyses of the present study.

#### Analyses

The statistical analyses concern drop-out and prediction. Drop-out analyses explored whether the patients who dropped out differed at baseline from study completers (patients who could be followed over a year). This was analyzed using two-tailed t-tests for independent samples for the continuous variables, two-tailed Mann–Whitney U-tests for the ordinal variables, and chi-square tests for the categorical variables.

The prediction analyses included a preliminary exploration of the relationships between the individual predictors and CBT outcomes, and a full exploration of the predictive power of the predictor set while controlling for pre-treatment score of the outcome measure and socio-demographic variables. For the preliminary exploration, a correlation matrix was composed. For the full exploration, hierarchical multiple regression analyses were used. In the first step of these regression analyses, pretreatment score on the outcome measure and socio-demographic variables were simultaneously entered as a block to statistically control for their impact on outcome. In the second step of these regression analyses, the predictors were simultaneously entered as a block to evaluate their impact as a set and as individual predictors on outcome. Since predictors have clinical relevance only if they are stable over time, these analyses were conducted for immediate, short-term and long-term CBT outcomes.

Five checks were used to verify whether the assumptions of hierarchical multiple regression analysis had been met and how accurate the resulting model was [33,39]. The first check used was Cook’s distance to explore whether the model was highly influenced by a small number of cases. The second check used was tolerance to confirm non-multicollinearity. The third check used was the Durbin-Watson statistics to confirm the independency of errors. The fourth check used was residual plots to explore for linearity and homoscedasticity. The fifth check used was the Shapiro-Wilk test to confirm the normality of standardized residuals.

## Results

### Patients

**Figure 1:**
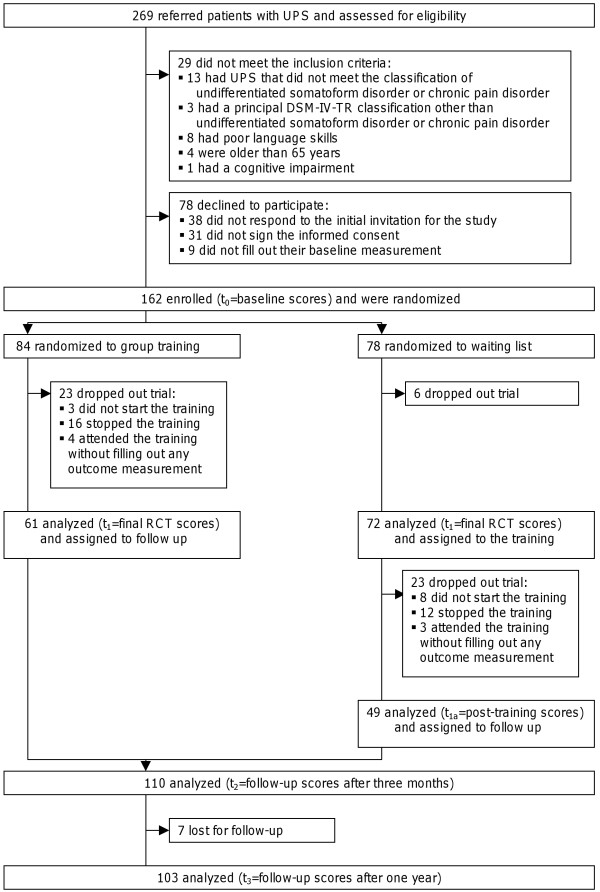
Patient flow.

Figure 1 shows the flow chart of patients through the study. The study started with 162 patients, 59 of whom dropped out.

The 59 patients who had dropped out of the study did not differ from the study completers with regard to their available scores for CBT outcome, control variables, and predictors (see Table 1), with the exception of a difference in the ‘age’ control variable. Patients who had dropped out were significantly younger (M=42.4, SD=11.1, p=.02) than the study completers (M=46.7, SD=10.8).

**Table 1:** Characteristics of CBT outcomes, control variables, and predictors

***CBT outcome***	**n**		
**Immediate CBT outcome, mean (SD)**	102	4.49	(7.59)
**Short-term CBT outcome, mean (SD)**	105	4.68	(6.79)
**Long-term CBT outcome, mean (SD)**	98	5.03	(7.55)
***Control variables***	**n**		
**Physical Component Summary - PCS, mean (SD)**	158	29.20	(8.97)
**Age in years, mean (SD)**	162	45.15	(11.05)
**Gender, n (%)**	162		
female		131	(81%)
male		31	(19%)
**Marital status*,*n (%)**	162		
married/living with a partner		110	(68%)
unmarried/divorced/widowed		52	(32%)
**Employment, n (%)**	162		
having employment		57	(35%)
having no employment		105	(65%)
***Predictors***	**n**		
**Global severity index, mean (SD)**	161	165.36	(50.28)
**Personality-disorder characteristics, mean (SD)**	160	15.07	(12.96)
**Psychiatric history, n (%)**	162		
presence of a psychiatric history		68	(42%)
absence of a psychiatric history		94	(58%)
**Mental Component Summary - MCS, mean (SD)**	158	45.15	(11.89)

With regard to the clinical characteristics shown in Table 2, no significant differences were found between the patients who had dropped out and the study completers.

**Table 2:** Clinical characteristics

**Clinical characteristics**	**n=162**
**Duration of UPS in years, median (interquartile range)**	9 (3–16)
**Classification of UPS by SCID-I**	
undifferentiated somatoform disorder	63
chronic pain disorder	99
**Number of comorbid DSM-IV Axis I disorders**	
no comorbid DSM-IV Axis I disorder	95
one comorbid DSM-IV Axis I disorder	43
two comorbid DSM-IV Axis I disorders	17
three comorbid DSM-IVAxis I disorders	4
four or more comorbid DSM-IVAxis I disorders	3
**Classification of comorbid DSM-IV Axis I disorder**	
mood disorder (in past; in lifetime)	24 (46;70)
anxiety disorder (in past; in lifetime)	47 (30;77)
substance-related disorder (in past; in lifetime)	1 (17;18)
eating disorder (in past; in lifetime)	1 (5;6)
psychotic disorder (in past; in lifetime)	0 (1;1)
other somatoform disorder (in lifetime)	26 (26)
adjustment disorder (in lifetime)	4 (4)
other disorder (in past; in lifetime)	1 (0;1)
**Number of comorbid DSM-IV Axis II disorders**	
no comorbid DSM-IV Axis II disorder	113
one comorbid DSM-IV Axis II disorder	27
two comorbid DSM-IV Axis II disorders	10
three or more comorbid DSM-IV Axis II disorders	10
**Classification of comorbid DSM-IV Axis II disorder**	
paranoid personality disorder	18
schizoid personality disorder	5
schizotypal personality disorder	2
anti-social personality disorder	1
borderline personality disorder	7
histrionic personality disorder	2
narcissistic personality disorder	2
avoidant personality disorder	29
dependent personality disorder	4
obsessive compulsive personality disorder	24
**Psychiatric history (number of *past *DSM-IV axis I disorders)**	
no past DSM-IV Axis I disorder	94
one past DSM-IV Axis I disorder	46
two past DSM-IV Axis I disorders	17
three past DSM-IVAxis I disorders	3
four or more past DSM-IVAxis I disorders	2
**Referrer**	
primary medical service	82
secondary medical service	51
secondary mental service	29

### Correlations between CBT outcomes, control variables, and predictors

Table 3 shows the correlation matrix between the CBT outcomes, the control variables, and predictors. With regard to the control variables, the only control variable that predicted outcome consistently over time was the pretreatment PCS score. A lower pretreatment PCS score was associated with a better CBT outcome. The pretreatment PCS scores explained five to nine per cent of the variance in CBT outcome. With regard to the predictors, no predictor predicted outcome consistently over time. The correlations with CBT outcomes were rather low according to Cohen’s guidelines [35]. Only the pretreatment MCS scores were significantly correlated with CBT short-term outcome. A higher pretreatment MCS score was associated with a better CBT short-term outcome. These pretreatment MCS scores explained six percent of the variance in the short-term CBT outcome.

**Table 3:** Correlation matrix with CBT outcomes, control variables and predictors

	**CBT outcome**		
	**Immediate**	**Short-term**	**Long-term**
***CBT outcome***			
Immediate CBT outcome	1.00		
Short-term CBT outcome	.60^***^	1.00	
Long-term CBT outcome	.58^***^	.58^***^	1.00
***Control variables***			
Physical Component Summary (SF-36: PCS)	-.30^**^	-.24^*^	-.22^*^
Age	-.03	.05	-.15
Gender	-.02	-.01	.04
Marital status	.07	-.02	.06
Employment	-.07	-.08	.07
***Predictors***			
Global severity index (SCL-90-R)	.11	.01	.10
Personality-disorder characteristics (VKP)	-.04	-.17	-.11
Psychiatric history (SCID-I)	-.01	.13	-.01
Mental Component Summary (SF-36: MCS)	.19	.25^**^	.11

^*^p≦.05, ^**^p≦.01, ^***^p≦.001.

### Prediction of immediate, short-term and long-term CBT outcome

Table 4 shows the hierarchical multiple regression models for predicting CBT outcome. The complete model was able to predict immediate CBT outcome (F(9, 92)=2.12, p=.04) and short-term CBT outcome (F(9,95)=2.85, p=.005); but not long-term CBT outcome (F(9, 88)=1.81, p=.08). When the effects of pretreatment outcome scores and socio-demographic variables were statistically controlled, the predictor set was only able to predict short-term CBT outcome (F(4,95)=4.41, p=.003); but not immediate CBT outcome (F(4,92)=0.17, p=.17) and long-term CBT outcome (F(4,88)=1.54, p=.20). The resulting explained variance in CBT outcome training was 6% at the end of CBT, 15% at three-month follow-up, and 6% at one-year follow-up. For short-term CBT outcome, better outcome was predicted by more psychological symptoms, fewer personality-disorder characteristics, the presence of a psychiatric history, and a better quality of life in the mental domain. There were no indications that the models were highly influenced by a small number of cases or by violating the assumptions of hierarchical multiple regressions analysis.

**Table 4:** Hierarchical multiple regression models for predicting CBT outcome

	**Immediate CBT outcome**^**a**^				**Short-term CBT outcome**^**b**^				**Long-term CBT outcome**^**c**^			
	**b**	**Standard error**	**β**^**I**^	**R**^**2**^	**b**	**Standard error**	**β**^**I**^	**R**^**2**^	**b**	**Standard error**	**β**^**I**^	**R**^**2**^
***Step 1***												
**Constant**	13.10	4.10			9.09	3.72			14.88	4.38		
**Control variables**												
Physical Component Summary (SF-36: PCS)	−0.27	0.08	-.34^**^		−0.19	0.08	-.26^*^		−0.22	0.08	-.27^*^	
Age	−0.05	0.07	-.07		0.03	0.07	.04		−0.11	0.08	-.16	
Gender	2.33	2.02	.12		1.09	1.79	.06		2.66	2.11	.13	
Marital status	1.60	1.65	.10		−0.49	1.51	-.03		1.39	1.66	.09	
Employment	−0.36	1.69	-.02		−0.16	1.51	-.01		1.26	1.73	.08	
Explained variance by control variables (R^2^)				.11				.07				.10
***Step 2***												
**Constant**	−10.06	10.22			−12.36	8.24			0.87	10.57		
**Control variables**												
Physical Component Summary (SF-36: PCS)	−0.16	0.09	-.20		−0.10	0.08	-.13		−0.15	0.09	-.19	
Age	−0.01	0.07	-.02		0.04	0.06	.06		−0.10	0.08	-.14	
Gender	1.05	2.20	.05		0.92	1.89	.05		1.51	2.32	.07	
Marital status	1.85	1.65	.11		−0.42	1.42	-.03		1.25	1.66	.08	
Employment	−0.53	1.68	-.03		−0.71	1.43	-.05		1.06	1.73	.06	
**Predictors**												
Global severity index (SCL-90)	0.06	0.03	.36^*^		0.06	0.02	.41^**^		0.06	0.03	.33^*^	
Personality-disorder characteristics (VKP)	−0.08	0.09	-.11		−0.19	0.07	-.32^*^		−0.18	0.09	-.28^*^	
Psychiatric history (SCID)	0.72	1.50	.05		2.79	1.27	.20^*^		1.12	1.52	.07	
Mental Component Summary (SF-36: MCS)	0.20	0.09	.30^*^		0.21	0.07	.36^**^		0.10	0.09	.15	
Explained variance by predictors ( ΔR^2^)				.06				.15^**^				.06

^a^ Immediate CBT outcome was computed by subtracting the baseline PCS-score from the post-training PCS-score.

^b^ Short-term CBT outcome was computed by subtracting the baseline PCS-score from the three-months follow-up PCS-score.

^c^ Long-term CBT outcome was computed by subtracting the baseline PCS-score from the one-year follow-up PCS-score.

^I^ β is the standardized value of b.

^*^p≦.05, ^**^p≦.01, ^***^p≦.001.

## Discussion

### Principal findings

We explored whether psychological symptoms, personality-disorder characteristics, psychiatric history, and health-related quality of life in the mental domain assessed at baseline predicted CBT outcome at the end of CBT (immediate outcome), three months after CBT (short-term outcome) and one year after CBT (long-term outcome), all after control for pretreatment scores on the outcome measure and for socio-demographic variables. We found that these predictors in combination with the control variables significantly predicted the immediate and short-term outcome of CBT, but not the long-term outcome.

The predictor set alone was significantly associated only with short-term CBT outcome. At this time-point, all predictors were significant. Psychological symptoms had the strongest association with short-term outcome followed by – in descending order of strength – health-related quality of life in the mental domain, personality-disorder characteristics, and psychiatric history. Psychological symptoms, health-related quality of life in the mental domain, and psychiatric history were positively associated with short-term outcome, meaning that a better outcome was expected if the number of psychological symptoms had been higher at baseline, if quality of life had been better at baseline, and if a psychiatric history had been present at baseline. Personality-disorder characteristics were negatively associated with short-term outcome, meaning a better outcome was expected if the number of personality-disorder characteristics had been lower at baseline.

As the predictor set did not significantly predict outcome at all three time points, its effects were not stable over time. This instability makes it unsuitable for selection and allocation of patients to CBT.

### Our principal findings in relation to the existing literature

The finding that effects of predictors were not stable over time was consistent with the findings of other studies [7-21] that have investigated the association between psychiatric comorbidity and outcome, and showed no stability of predictors between studies or over time within studies. However, unlike these studies [10,12,15], our own study controlled statistically for the effects of pretreatment score on the outcome measure and socio-demographic variables. Even under these conditions, predictors still did not consistently predict which patients benefited from CBT and which did not.

The possible reasons for the inconsistent findings over studies on predictor effects might be that the study groups were too small or the group of patients with UPS studied was too heterogeneous. Other reasons might be that the sets of predictors in the studies were wrongly measured and/or chosen. Predicting CBT outcome might be more complex, and may require a larger set of variables than the sets of predictors which were investigated in the studies. For instance, it may require a good match between trainer and trainees, a supportive but not over-protective partner, no deaths nearby, no moving house, and no termination of employment. It has been estimated that factors in client-therapist relationship account for 30% of treatment outcome, and factors outside CBT for another 40% [40].

### Strengths and limitations in the study

The strength of the present study was that the impact of a predictor set on the primary outcome measure was explored at three time points over one year after CBT [2]. As most other studies were designed to predict treatment outcome at only one time point after CBT [8,13-15], they did not explore the stability of the predictor effect over time. They also used more than one outcome measure without indicating the primary outcome measure [8,12,15,18]. By this, other studies did not explore the stability of predictor effects over time on the outcome measure that was preliminary chosen as the most important outcome of CBT.

A limitation of the study was that personality-disorder characteristics were measured using a self-report questionnaire. Although many patients met the DSM-IV axis II criteria for the classification of a specific personality-disorder subtype, only two met the general criteria applying to all personality disorders. Patients seemed to view any maladaptive thoughts, feelings and behavior associated with personal and social disruption as a specific reaction to a situation rather than a pervasive and stable pattern across many situations. If these reactions were indeed determined by a specific situation, our classification of their personality disorders would have been incorrect. However, a high number of personality disorders was to be expected: our patients had had their UPS for an average of nine years, and the prevalence of personality disorders in such patients is four times higher than in the healthy population [41]. The total number of DSM-IV axis II criteria confirmed in our study (mean=15.07 and standard deviation=12.96) was significantly larger than the mean in the ‘normal population’ reference group (mean=12.54 and standard deviation=9.79), and significantly less than the one in the ‘psychiatric patients’ reference group (mean=25.37 and standard deviation =13.55) [32]. The validity problem caused by failure to meet the general criteria applicable to all personality disorders might be partially solved by using structured interviews. Although this might provide a more objective perspective, it still depends on information communicated by patients and also on patients’ views of their own thoughts, feelings and behavior.

### Clinical and policy implications

In routine practice assessments, patients with UPS are selected and allocated to different kinds of treatment on the basis of psychological symptoms, personality-disorder characteristics, psychiatric history, and health-related quality of life. As the prevalence of comorbid mood, anxiety [42], and personality disorders [41] is high in this patient group, patients are often redirected from treatment for UPS to treatment for mental health problems, which is usually provided by the mental health services. However, these predictors did not consistently predict CBT outcome, neither in our study nor in our review of other studies, and as a substantial number of patients communicates in physical and not mental terms [43] and refuses to be referred to the mental health services [5,44], this practice does not seem to be appropriate.

## Conclusions

As psychological symptoms, personality-disorder characteristics, psychiatric history, and health-related quality of life in the mental domain assessed at baseline did not seem to predict CBT outcome consistently over time, the need for selection and allocation of patients on the basis of these variables to CBT is doubtful. In our study, if patients had been excluded from CBT on the basis of these variables, they would have been deprived unnecessarily of effective group training.

## Abbreviations

CBT: Cognitive behavioral therapy; MCS: Mental component summary of the SF-36; PCS: Physical component summary of SF-36; SCID-I: Structured clinical interview for DSM-IV axis I disorders; SCL-90-R: 90-Item symptom checklist revised; SF-36: 36-item medical outcomes study short-form general health survey; UPS: Unexplained physical symptoms; VKP: Vragenlijst kenmerken van persoonlijkheid, a Dutch self-report questionnaire based on the international personality disorder examination.

## Competing interests

All authors declare that they have no competing interests by publishing this report.

## Authors’ contributions

LNLZ developed the original idea of the study, implemented the study, conducted the analyses and drafted the manuscript. All authors read and corrected draft versions. All authors approved the final version of this manuscript.

## Authors’ information

LNLZ is clinical psychologist, psychotherapist and cognitive behavioral therapist. YRvR is clinical psychologist, psychotherapist and cognitive behavioral therapist. CGK is psychiatrist and psychoanalytist. RT is assistant professor and statistician. AvtS is assistant professor and psychotherapist. JJVB is professor of health-related quality of life, ad interim chair of the department Medical Psychology and Psychotherapy of Erasmus Medical Center in Rotterdam, and managing director of the Viersprong Institute for studies on Personality Disorders (VISPD) of the ‘Viersprong’ in Halsteren, the Netherlands.

## Pre-publication history

The pre-publication history for this paper can be accessed here:

http://www.biomedcentral.com/1471-2458/12/848/prepub
